# The future of quality improvement research

**DOI:** 10.1186/1748-5908-8-S1-S9

**Published:** 2013-04-19

**Authors:** Rebecca S Miltner, Jeremiah H Newsom, Brian S Mittman

**Affiliations:** 1 School of Nursing, University of Alabama at Birmingham, Birmingham, Alabama, 35294, USA; 2 Department of Hospital Medicine, Ochsner Health System, New Orleans, Louisiana, 70121, USA; 3 VA Center for Implementation Practice and Research Support, VA Greater Los Angeles Healthcare System, Los Angeles, California, 91343, USA

## Presentation

The history of quality improvement research (QIR) demonstrates the large growth in improvement activities from early quality assessment and small area variation work through the adoption of industrial quality improvement methods in healthcare operations to the recent opportunities inherent in the Affordable Care Act of 2010. But after 40 years of development, significant growth in these scholarly activities has not produced comparable growth in insights, practical guidance, or progress toward better care.

Five challenges influence the trajectory of improvement work and implementation science. The first challenge relates to the innovations and evidence base to improve healthcare. The focus of innovation and research in improvement has been on strategies, facilities and systems that are leaders in performance and quality improvement – the organizational equivalents of healthy white males. This makes differentiation and generalizability to the range of organizational settings difficult. A concerted effort must be made to focus on research conducted within, and with relevance to, the broader practice environment.

The second challenge includes multiple logistical barriers such as access to study sites, the limited funding opportunities for QIR, and the lack of consistent IRB guidance and interpretation of regulations. Underlying these barriers is a considerable lack of clarity surrounding the nature of QIR relative to other types of health research. With the exception of the recent statement on cluster randomized trials by the Ottawa Ethics of Cluster Randomized Trials Consensus Group (2012)[1], the absence of consistent guidelines for QIR poses challenges for researchers trying to obtain local IRB review as well as investigators competing with others using more traditional research methods during the grant review process. QIR researchers need to embrace the IRB process and develop explicit, consensus-based guidance to facilitate more consistent reviews at the funding and IRB stages.

The third challenge includes the professional differences that stem from the diversity of academic disciplines and types of institutions from which people involved in this work emerge. The concepts and definitions arising from diverse disciplinary roots make it difficult to achieve progress and move forward collectively. These factors pose barriers to the scholarly QIR community and, more importantly, contribute to confusion and decreased credibility among external stakeholders and scientists in the more traditional fields of study surrounding QIR. Lack of consensus and clarity impede the advance of this science because researchers cannot explain the work consistently to funding agencies, editorial review boards and other stakeholders.

The fourth challenge is the need to strengthen the theoretical foundations for this work. There is an urgent need to assess whether we have the right theories, too many theories or simply a lack of guidance in using theories to build the science of improvement.

The weak theoretical basis for QIR contributes to the fifth and final challenge to the future, which is advancing the science through robust and appropriate research approaches, designs and methods. The field has failed to reach consensus about the major research questions and goals for QIR, and continues to debate the appropriate methods for improvement and implementation work. Different views regarding the value and need for various research approaches and methods for conducting QIR limits the production of practical and effective insights and tools for researchers, clinicians, organizations, and policy decision makers.

## Commentary

The challenges were corroborated by other expert presenters reported in these proceedings. The field is at a crossroads and facing the need to agree on the correct path to resolve the critical challenges for future success. Professionals in this field bring multiple perspectives which are both opportunities and barriers to advance the goals, scope of work, key concepts and definitions for QIR for the future. Foundational elements are essential to move improvement science forward. A lack of consensus adds to the tension about the core of QIR. For example, 150 conference participants involved in improvement science did not agree on what improvement research means. Some described improvement research as the study of the effectiveness of quality improvement tools and methods. Others included measurement and implementation issues and the study of processes and mechanisms of practice change as key questions in the field of improvement science. The gaps in consensus were evident in the inability to identify similarities and difference between improvement and implementation science. The inability to clearly define quality improvement research and improvement science impedes the future success of QIR.

QIR scholars need to define this scientific field and its goals or others will define it for us. At the International Forum on Quality & Safety in Healthcare in 2012, Paul Batalden suggested that improvement science is useful knowledge to improve health care that is needed to both discover and assess what is effective in practice[2]. Bataldan’s view is probably the most consistent with conference attendees’ current view of the science. Blumenthal recently suggested national performance improvement activities with a focus on cost containment looking at high cost patients with multiple chronic conditions using tools of primary care, payment reform and better information [3]. Berwick and Hackbarth charge that addressing waste is critical to reducing costs, improving care and creating sustainable healthcare for all [4]. These larger program mandates provide an opportunity for future improvement science and implementation science work. But the focus on national large-scale improvement initiatives tends to undervalue the daily local improvement work done by interprofessional teams within a microsystem. Integrity of improvement science is needed at the micro and macro system levels to successfully deliver safe, effective and efficient care for all.

## Recommendations

Questions directed to conference presenters centered on what needs to occur next in order to move improvement science forward. The current trend of rather laissez faire, unfocused scientific development in the field has been beneficial as it has allowed researchers to explore new ideas and learn from a diverse array of approaches. But at this time, QIR needs to build a stronger science to improve healthcare on a larger scale. Other fields of study have struggled with the same types of conceptual, theoretical and methodological issues, and these fields have benefited from concerted efforts to address these questions. The improvement science community needs to take a systematic approach to defining its scope, developing its theoretical base and strengthening the research tools required for progress. Basic consensus on common scientific language and research approaches is needed to successfully design studies, move efficiently through IRB processes and peer review, and communicate with organizational leaders and health policy decision makers.

Conference attendees agreed that the Academy for Healthcare Improvement is well-placed to convene a task force to conduct this critical foundational work. Action is needed to strengthen and guide this specialized area of science to develop expertise and interest by attending to basic definitions of what QIR is, what the scope of our work includes, and how implementation and improvement sciences intersect. Funding opportunities from both public and private sources are needed to support a rich array of improvement projects, but this will require that researchers work consistently from a solid conceptual base. This basic work is essential to set a strong course for the future of quality improvement science.

## References

[B1] Weijer C Grimshaw JM Eccles MP McRae AD White A The Ottawa statement on the ethical design and conduct of cluster randomized trials PLoS Med 2012 9 11 e1001346 10.1371/journal.pmed.1001346 23185138 PMC3502500

[B2] Batalden P Improvement Science Symposium 2012 2012 International Forum on Quality and Safety in Healthcare. Paris, France

[B3] Blumenthal D Performance improvement in health care—Seizing the moment NEJM 2012 366 21 1953 1955 10.1056/NEJMp1203427 22533534

[B4] Berwick DM Hackbarth AD Eliminating waste in US health care JAMA 2012 307 14 1513 1516 10.1001/jama.2012.362 22419800

